# Occurrence, Distribution, and Risk Assessment of Perfluoroalkyl Acids (PFAAs) in Muscle and Liver of Cattle in Xinjiang, China

**DOI:** 10.3390/ijerph14090970

**Published:** 2017-08-28

**Authors:** Gehui Wang, Jianjiang Lu, Zhenni Xing, Shanman Li, Zilong Liu, Yanbin Tong

**Affiliations:** School of Chemistry and Chemical Engineering, Shihezi University, Shihezi 832003, China; wang_ge_hui@163.com (G.W.); xzn_xingzhenni@163.com (Z.X.); lishanman8674@sina.com (S.L.); liuzilong1999@163.com (Z.L.); tongyanbin@sina.com (Y.T.)

**Keywords:** perfluoroalkyl acids, muscle, liver, average daily intake, health risk, Xinjiang

## Abstract

Despite risks associated with perfluoroalkyl acids (PFAAs) in many regions, little is known about their prevalence in Xinjiang. We determined the presence of 13 PFAAs in 293 beef muscle and liver samples collected in 22 cities covering northern, southern, and eastern Xinjiang using liquid chromatography, coupled with tandem mass spectrometry. Overall, the average values for PFAAs were relatively low compared with previous studies. Liver presented higher mean levels of total PFAAs at 1.632 ng/g, which was over 60-fold higher than in muscle (0.026 ng/g). Among the PFAAs analyzed, medium-chain compounds were dominant, accounting for more than 70% of the total. Perfluorooctane sulfonate (PFOS) was highly prevalent in the liver with the highest mean concentration (0.617 ng/g) and detection frequency (80%). When comparing the three regions of Xinjiang, we found differences in PFAA profiles, with the northern region showing the highest levels. Furthermore, the average daily intake and hazard ratios of PFOS and perfluorooctanoic acid varied by region, urban/rural environment, gender, ethnicity, and age. The highest risk value of 13 PFAAs was estimated to be 0.837 × 10^−3^, which is far below 1, indicating that there is no health risk posed by eating beef muscle and liver in Xinjiang.

## 1. Introduction

Perfluoroalkyl acids (PFAAs) are an anthropogenic, emerging class of persistent organic pollutants (POPs). In general, these compounds are composed of a fully fluorinated carbon chain with an acidic terminal group, such as a carboxylic, phosphonic, or sulfonic acid moiety, with the strong chemical bonds of C-F conferring their unique amphiphilicity, persistence, and bioaccumulation properties [1,2,3]. Consequently, PFAAs have been employed in a wide variety of industrial and commercial products, such as insecticides, lubricants, coatings, paper treatment, food packaging, fire-fighting foams, fabrics, and carpets [3,4,5].

Large quantities of PFAAs have been manufactured and used since the 1950s, representing more than 60 years of existence. The 3M Company declared a phase-out of perfluorooctane sulfonate (PFOS) and perfluorooctanoic acid (PFOA) (the two most frequently used PFAAs) and their long-chain homologs in 2000 due to their widespread contamination and potential risks to the environment and to humans, and then phased-in a series of shorter-chain alternatives with relatively low toxicity [2,3]. In recent years, the production and application of PFAAs-based products have been discontinued and restricted in a number of developed countries; however, in China, the use of PFAAs-related chemicals has continued to increase to meet ongoing domestic and overseas industrial demand [6].

Because of their diffuse sources and exceptional stability, PFAAs appear in a variety of environmental habitats and are distributed worldwide in humans. Their toxic effects on the human body have been confirmed in numerous mammalian toxicology studies and in general population epidemiological investigations [7,8,9]. Therefore, the occurrence of PFAAs in humans has attracted great attention in recent years [7,10,11,12], as have the exposure routes (water, air, house dust, and food) [13]. Comparing the main sources of human exposure, D’Hollander et al. [14] found that food consumption is the most important. Similar results were obtained from a Swedish study [15], which showed that human exposure to PFAAs via dietary intake is about 6–10-fold higher than through dust or water. Food intake has been identified as the predominant pathway of human exposure [16,17]. Therefore, in the last several years, the occurrence of PFAAs in a diverse range of foods has been evaluated in many countries, such as the USA, European countries, Japan, Korea [4,18,19,20,21], and China [22,23]. In these studies, relatively high PFAA levels often correspond to animal-derived foods (especially meat, fish, shellfish, and dairy). The measurement of 21 perfluoroalkyl substances in animal-derived foods originating from four European countries [18] showed concentrations of individual compounds ranging from 0.002 to 0.076 ng/g. The occurrence of 16 PFAAs in 397 foods (66 types) was analyzed in Korea, and meat products were found to contain high mean levels of 1.610 ng/g [21]. Risk assessments of these compounds associated with diet have been reported worldwide, and fortunately, the average daily intake (ADI) is calculated to be far below the existing tolerable levels set by the European Food Safety Authority [4,15,18,21]. However, these substances are bioavailable and can accumulate in the environment and food chain, and the long-term risks they present to humans should be given more attention. It should be mentioned that monitoring target compounds tends to be limited to PFOS and PFOA, and hence, the risk posed by other PFAAs cannot be ignored.

Despite the most well-known PFAAs having been investigated in meat products in many previous studies, there is little knowledge of their fates and behaviors in Xinjiang, which lies in the northwest frontier of China and is a region dominated by animal husbandry. Beef is one of the most popular meat products, especially for the local Uyghurs. Moreover, a fragile environment coupled with unique climatic conditions in this region may play an important role in exacerbating contamination by PFAAs [5]. Therefore, 13 representative PFAAs including long chains, medium chains and short chains were selected in this study, and the main objectives were (i) to analyze the behaviors of these substances in beef muscle and liver sampled in Xinjiang; (ii) to investigate the spatial distributions of PFAAs and to identify the sources of pollution; and (iii) to estimate the daily dietary intake of PFAAs via beef muscle and liver to assess the health risks they pose to the Xinjiang population.

## 2. Materials and Methods

### 2.1. Sampling

**Figure 1:**
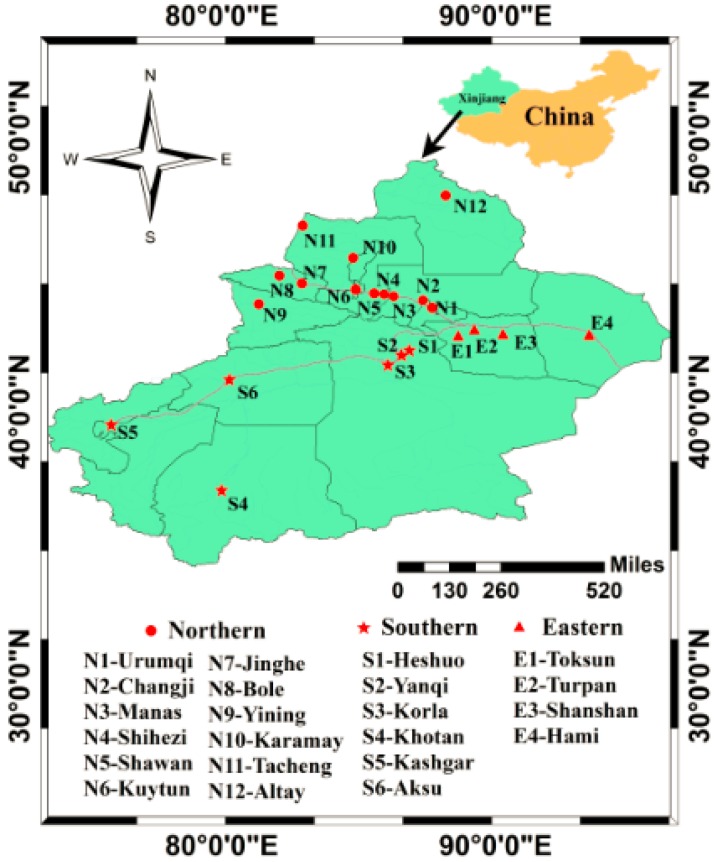
The distribution of sampling sites in northern, southern and eastern Xinjiang.

Beef muscle (*n* = 176) and beef liver (*n* = 117) were randomly purchased from local supermarkets, retail stores, farmers markets, or farms of 22 cities in Xinjiang from November 2015 to September 2016. The sampling points represented the major cities of northern (N1–N12, *n* = 157), southern (S1–S6, *n* = 80), and eastern (E1–E4, *n* = 56) parts of Xinjiang (Figure 1). When the samples were collected, some information on the cattle was gathered such as breed, sex, age, feeds, feeding modes, and whether it was produced locally. According to the information collected, 31 out of 176 muscle samples were collected from home-raised cattle, and 81 were from commercial feed cattle, and the other 64 samples had no exact feeding information. All the samples were packaged by aluminum foil and kept refrigerated before rapid transport to the laboratory, and then stored at −40 °C until chemical analysis.

### 2.2. Chemicals and Standards

All the analytical standards used in present study were obtained from Wellington Laboratories (Guelph, ON, Canada). Native standards were: perfluorobutanoic acid (PFBA, 98%), perfluoropentanoic acid (PFPeA, >98%), perfluorohexanoic acid (PFHxA, 98%), perfluoroheptanoic acid (PFHpA, 99%), perfluorooctanoic acid (PFOA, 95%), perfluorononanoic acid (PFNA, 97%), perfluorodecanoic acid (PFDA, 97%), perfluoroundecanoic acid (PFUdA, 95%), perfluorododecanoic acid (PFDoDA, 96%), perfluorotridecanoic acid (PFTrDA, 97%), perfluorobutane sulfonate (PFBS, 97%), perfluorohexane sulfonate (PFHxS, ≥98%), and perfluorooctane sulfonate (PFOS, ≥98%). Labeled standards used were: perfluoro-n-[1,2,3,4-^13^C_4_]-octanoic acid (MPFOA, >99%, 1,2,3,4-^13^C_4_); Sodium perfluoro-1-[1,2,3,4-^13^C_4_]-octane sulfonate (MPFOS, >99%, 1,2,3,4-^13^C_4_). Tetrabutylammonium hydrogen sulfate (TBA, HPLC-grade) and tert-butyl methyl ether (MTBE, chromatographic grade, ≥99.9%) were supplied by Aladdin (Los Angeles, CA, USA). Methanol (MeOH, HPLC-grade) and acetonitrile (ACN, HPLC-grade) were from Fisher Scientific (Pittsburgh, PA, USA). Ammonium acetate (NH_4_OAc, HPLC-grade, 99.99%) was supplied by Sigma-Aldrich (St. Louis, MO, USA). Sodium carbonate anhydrous (super grade, >98%) and sodium hydrogen carbonate (super grade, ≥99.8%) were purchased from J&K (Beijing, China). Oasis HLB (6 cc, 200 mg) was provided by Waters (Milford, MA, USA). All experimental water used in this study was ultrapure water (>18.2 MΩ/cm) prepared in the laboratory.

### 2.3. Sample Preparation

The target compounds were extracted from muscle and liver by an ion-pairing method published previously [24,25,26], with some modifications. Thawed muscle and liver samples were homogenized, then freeze-dried for at least 12 h and ground into powder before extraction. First, 0.3 g of each sample was placed into a 15 mL polypropylene (PP) centrifuge tube, then, 2 ng internal standards (MPFOS and MPFOA) were added before adding 1 mL of TBA solution (0.5 mol/L) and 2 mL of 0.25 mol/L sodium carbonate buffer (pH = 10). Subsequently, 5 mL of MTBE was used for extraction followed by shaking for 20 min at 250 rpm. Then, the solution was centrifuged for 10 min at 6000 rpm to separate the organic and aqueous layers, and the supernatant (MTBE) was collected in another PP tube. The extraction was repeated once more and the two extracts were combined. All extracts were concentrated to a volume of 0.5 mL under nitrogen gas, and diluted with ultrapure water to 40 mL in a PP tube loaded onto a HLB single-use cartridge. This cartridge was pre-conditioned by MeOH (5 mL) and ultrapure water (7 mL), and washed with 5 mL 10% methanol solution (MeOH/H_2_O, 1/9, *v*/*v*). After that, it was dried for 30 min under a vacuum and eluted with 7 mL MeOH. Throughout the treatment process, the velocity of flow was 1–2 drops per second. The obtained eluent was then blown to near dryness by nitrogen and reconstituted in 1 mL of mobile phase (ACN/10 mM NH_4_OAc, 40/60, *v*/*v*). The reconstituted extract was filtered into an autosampler vial using a 0.22 μm nylon syringe filter prior to the final HPLC-MS/MS analysis. Eventually, the concentration of dry weight was converted to wet weight through the ratio of dry and wet weights.

### 2.4. Instrumental Conditions for HPLC-MS/MS

In this study, HPLC-MS/MS analysis was performed for 13 target PFAAs. A total of 10 μL sample was injected into an Agilent 1200 series HPLC system (Agilent Technologies, Santa Clara, CA, USA) equipped with an automatic degasser, a quaternary pump, and a thermostated column compartment (maintained at 40 °C). Separation of target compounds was achieved on an Zorbax Eclipse Plus C_18_ 80A column (2.1 × 100 mm, 3.5 μm, Agilent Technologies). The mobile phase consisted of 10 mM NH_4_OAc aqueous solution (A) and 100% ACN (B). During the process of gradient elution, the initial stage was 60/40 A/B followed by 9 min ramped to 10/90 A/B and held for 3 min. Ultimately, it was reconditioned for 8 min of stabilization time using the initial conditions (60/40 A/B). Detection was performed with an Agilent 6410 triple-quadrupole mass spectrometer that operated in negative ion multiple reaction monitoring (MRM) mode. Further details, such as monitored MS/MS transitions, retention time (RT), and collision energy (CE) are given in the supplementary information (Table S1).

### 2.5. Quantification and Quality Control

Quantification was performed by adding the isotopically labeled standards (^13^C_4_-PFOA, ^13^C_4_-PFOS) into all samples at the beginning of extraction. Therefore, errors such as matrix interferences, extraction and/or MS acquisition discrepancies could be quantified and corrected. A 10-point matrix-matched calibration curve with concentration levels from 0.01–20 ng/mL was prepared using blank matrix extract serial dilutions for each analyte, and all correlation coefficients (R^2^) were better than 0.990. The limits of detection (LODs) for each substance were estimated as the concentration with a signal-to-noise ratio (S/N) of 3, and the limits of quantification (LOQs) were defined as the lowest calibration standard with S/N higher than 10. In addition, recovery tests for all PFAAs at three spiking levels (0.02, 0.5, 1 ng/g) to blank matrices were performed. The LODs for muscle varied in a range of 0.012–0.06 ng/g; mean recoveries were between 73.8 and 115.4%. For liver, the LODs ranged from 0.003 to 0.036 ng/g, and mean recoveries were between 84.2 and 110.0%. Relative standard deviations (RSDs) repeated for six extractions were lower than 10%, indicating that the precision of the method was good. More detailed information (linear equation, LODs/LOQs, recovery, and RSDs) of the 13 measured PFAAs and 2 internal standards provided in supplementary information (Table S2).

Before and after detecting a batch of 20 samples, calibration standards were injected to check instrument response to ensure that the standard response deviation was less than ± 20%. Methanol solution (instrumental blanks) was injected every 10 samples to eliminate memory effects. To monitor contamination of reagents and glassware, procedural blanks were injected every 20 samples. New PP centrifuge tubes were used in all experiments and all materials with polytetrafluorethylene (PTFE) were removed from the experimental stations. All test containers were alternately rinsed twice with ultrapure water and MeOH before use. The instrument was rinsed overnight before use, and the ion source was cleaned regularly. All experimental results were deducted from blank experimental data.

### 2.6. Daily Intake Estimation and Risk Assessment

To elucidate the contribution of beef muscle and liver consumption to human exposure to PFAAs, we evaluated the daily intakes of PFOS and PFOA (the two most frequently found analytes) and the possible health risks caused by them. Muscle consumption data were obtained from a survey on dietary structure analysis in Xinjiang [27], conducted in 2010. The calculations were performed based on in-person interviews and questionnaires of 3000 adults, aged 18 to over 70, gaining their dietary information over the past year. Additionally, the consumption data for the liver was calculated by the ratio of liver weight to muscle weight due to a lack of data on Chinese daily intake values, a method also applied by Zhang et al. [22]. A recently published study from Xinjiang focused on the relationship between health risk and milk intake, and the average body weight (60 kg) was used in this study [5].

The daily intake (DI) is the average daily consumption for muscle and liver per unit body weight, ng/kg/day, and the calculation is the sum of daily consumption values of muscle and liver (CD_muscle/liver_) divided by the average body weight (BW), as shown in Equation (1). The hazard ratios (HR) for PFOA or PFOS were calculated with their respective mean concentrations in the samples (C_PFOS/PFOA_) multiplied by DI (the product is ADI, ng/kg bw/day) and divided by their reference dose (Rfd; Rfd_PFOA_: 1500 ng/kg bw/day, Rfd_PFOS_: 150 ng/kg bw/day) [21], which are listed in Equations (2) and (3). When HR > 1, it indicates that there is potential risk of human exposure to PFAAs; otherwise, there is low potential health hazard. Concentrations below the LOD, were treated as zero and recorded as ND.

The estimated formulas were as follows:

(1) $$\mathrm{DI}=(CD_{\mathrm{muscle}}+CD_{\mathrm{liver}})/\mathrm{BW},$$

(2) $$\mathrm{ADI}=C_{\mathrm{PFOS}/\mathrm{PFOA}}\times \mathrm{DI},$$

(3) HR=ADI/Rfd

## 3. Results and Discussion

### 3.1. PFAA Concentrations across Beef Muscle and Beef Liver in Xinjiang

The aggregated data of 13 measured PFAAs are presented in Table 1. In muscle, 83 out of 176 (47%) samples had detectable levels of PFAA, and 110 out of 117 (94%) in liver. The concentration ranges of individual compounds in these two foods were: ND–0.103 ng/g for muscle and ND–4.221 ng/g for liver, and the mean level of total PFAAs (∑PFAAs) in muscle (0.026 ng/g) was over 60-fold lower than in liver (1.632 ng/g). PFOA was detected in 24% of the muscle samples and in 75% of liver samples; as for PFOS, it was detected in 30% and 82% of muscle and liver samples, respectively. These two substances were the most frequently detected PFAAs. PFOS (mean: 0.617 ng/g) showed marked accumulation potential in liver compared with PFOA (0.264 ng/g) (Table 1), and similar results have been obtained in many other studies [24,28,29].

For muscle samples, only PFOS, PFOA, PFHpA, and PFUdA were above their respective LODs, and all the detection frequencies were lower than 50%. However, nearly all target compounds were found in liver except for PFHxS (under its LOD) (Table 1). A discovery by Kowalczyk et al. [29] showed that PFHxS has a high accumulation in cows, however it was not detected in the present study, which indicated that the pollution level of PFHxS is extremely low or there are no direct pollution sources in Xinjiang. In addition to PFOS and PFOA, PFUdA, PFDA, and PFNA were more prevalent compounds in liver and their frequencies were 73%, 58%, and 56%, respectively. In addition, PFHxA and PFBS were also detected in a few liver samples (12% and 19%), and the emergence of PFBS, which can be easily eliminated by urine, may be related to the use of new fluorinated materials as chemical replacements in Xinjiang [4,29]. PFHxA may result from cross-contamination from contact with materials when transported or manipulated [4]. Other contaminants, such as PFPeA, PFDoA, and PFHxA, were only detected at 3%, 9%, and 12% from the liver samples, respectively (Table 1).

### 3.2. Regional Differences of PFAAs in Xinjiang

**Figure 2:**
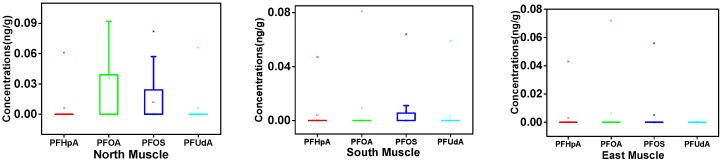

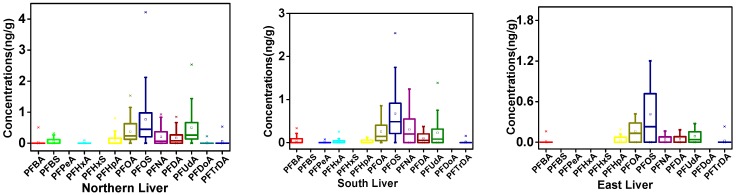
Box-and-whisker plots of detected PFAAs in muscle and liver from southern, northern and eastern Xinjiang (ng/g, wet weight). The horizontal line and the small square in the box respectively represent the mean and median value, and the 25th and 75th percentiles are marked by the lower and upper edges of the box. The whiskers extending from the box represent the highest and lowest values and the asterisks show extreme values.

We investigated variations in PFAA levels among northern, southern, and eastern Xinjiang, and these are displayed in Figure 2. The highest mean ∑PFAAs concentration (muscle: 0.042 ng/g, liver: 2.330 ng/g) was observed in northern Xinjiang followed by the south (0.023 ng/g, 1.705 ng/g) and east (0.014 ng/g, 0.819 ng/g) (Figure 2). Regarding the detection frequencies of each compound, the results agreed with the order of north > south > east. In addition, 12 of 13 PFAAs were detected in the north, followed by 10 in the south and 8 in the east. Northern Xinjiang, possessing the largest population, is more urbanized and industrialized than the south and east, which is assumed to lead to relatively higher PFAA exposure [5,30]. In addition, a higher precipitation rate in northern Xinjiang [31] may be another cause. It has been reported that water deposition is a major pathway for PFAAs from the atmosphere to other environmental matrices, including soil, water, and even food [32]. The southern region is mainly engaged in agriculture, and the extensive use of pesticides will contribute to exposure to PFAAs in this region [6]. In addition, more chemical and petroleum industries are emerging in the south, and this may explain the higher PFAA exposure in this area compared with eastern Xinjiang.

**Figure 3:**
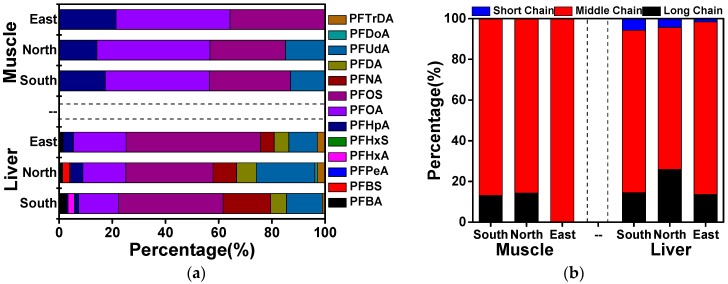
Composition profiles in muscle and liver samples of southern, northern and eastern Xinjiang. (**a**) the 13 detected PFAAs; (**b**) long chains (C11–C13), medium chains (C7–C10) and short chains (C4–C6).

Similar PFAA profiles were observed in the same food items (Figure 3a). PFOA and PFOS constituted a notably high proportion (49–79%) of ∑PFAAs in the two food groups among the three parts of Xinjiang. PFUdA was another prevalent compound, constituting up to 22% of the total in livers of the northern region. Similar values for PFNA and PFDA were observed in northern and eastern Xinjiang. A higher proportion of ∑PFAAs corresponded to PFNA (22%) in the south, whereas PFDA accounted for fewer than 7% of the total, suggesting that different sources for these two substances probably exist in the southern region. As shown in Figure 3b, medium-chain compounds were the predominant contributors in all samples accounting for approximately 70–100% of ∑PFAAs. Short-chain PFAAs were non-existent in muscle samples, but observed in liver accounting for less than 10% of the total. Long-chain PFAAs in northern Xinjiang accounted for 26% of the total. However, lower proportions of less-than 15% were found in the southern and eastern regions, perhaps because extra sunlight leads to direct photolysis of long chain compounds [32,33]. In addition, the intense ultraviolet radiation can also degrade long-chain PFAAs into medium-short substances [34].

### 3.3. Comparison with Recent Studies of PFAAs in Animal Samples

**Figure 4:**
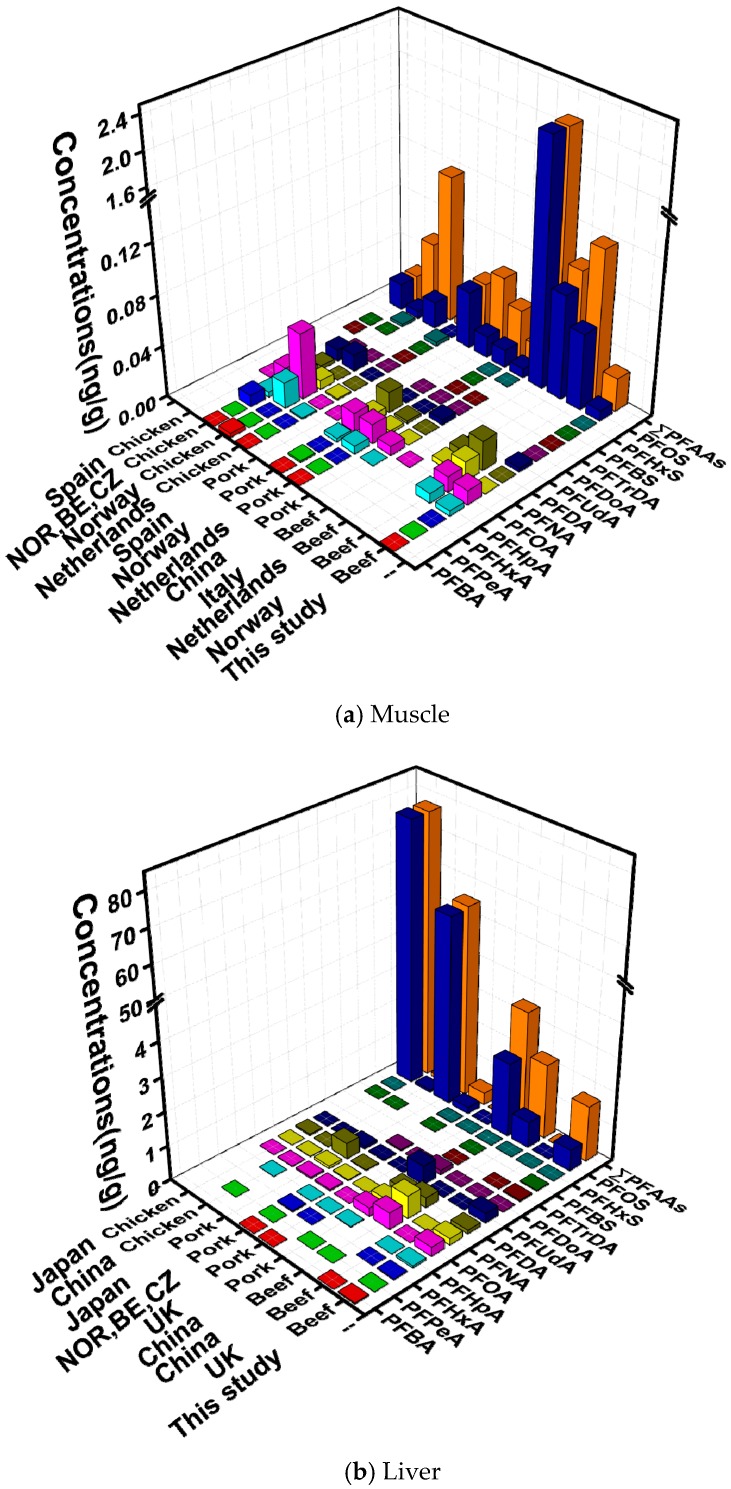
Comparison of the mean concentrations (ng/g, wet weight) of PFAAs with other recent studies of animal muscle and liver samples (NOR, BE, CZ: Norway, Belgium, Czech Republic; no bar: no corresponding values; [16,22,36,37,38,39,40,41]).

A summary of recent studies (2008–2014) of individual compounds in animal muscle and liver is given in Figure 4. In general, our results (muscle: 0.026 ng/g; liver: 1.632 ng/g) were lower than those found in other countries such as The Netherlands (beef meat: 0.094 ng/g), Italy (beef meat: 2.11 ng/g), other European countries (beef meat: 0.08 ng/g) [13,22,35,36], and China (beef meat: 4.43 ng/g, beef liver: 2.18 ng/g) (Figure 4). The relatively low concentrations we found are largely due to the lack of PFAAs-using industries [7]. Among the analyzed PFAAs, PFOA and PFOS were predominant compounds in all studies. In addition, PFNA, PFDA, and PFUdA were also relatively abundant (Figure 4).

When comparing PFAA contaminations among different animal matrices, higher levels were observed in beef muscle, whereas beef liver had lower levels than chicken and pork (Figure 4). Differences in concentrations of pollutants may be related to the sampling from different animals or different kinetic patterns for PFAAs. A higher accumulation tendency of PFOS was shown in male chickens compared with cows and sheep [29,42]. Additionally, the kinetic pattern of each compound also differs, even in identical animal bodies [43].

Large spatial variations of PFAAs levels are observed among countries. For muscle samples, those collected from Italy showed the highest concentrations (beef meat: 2.11 ng/g) [36]; for liver, the highest levels were observed in Japan (chicken liver: 77.04 ng/g) [41] (Figure 4). Many factors could contribute to these spatial differences, primarily differences in sampling time and sites. A study carried out to evaluate the dietary exposure to 11 PFAAs in Sweden [15] used food samples from 1999, 2005, and 2010, and inter-year differences were demonstrated especially in egg and meat samples. The economic development of sampling sites is also closely associated with contamination by PFAAs [5,30]. In addition, the age of the animal at the time of slaughter is another factor. The higher levels of PFOS (pig: 54 ng/g, chickens: 77 ng/g) in Japan might be related to the elder age and longer growth time (pig: 7 months, chickens: 2.5–7 months) compared with Beijing (chickens: 0.063 ng/g, 40 d; pigs: 2.163 ng/g, 5 months) [23,26]. In addition, the differences in living and eating habits of animals also explain the spatial distributions [39].

### 3.4. Source Analysis of PFAAs in Cattle of Xinjiang

Similar to human exposure pathways, cattle may be exposed to PFAAs by ingesting food, drinking water, and inhaling air/dust. It has been found that PFOS and PFOA can be transferred from soil to crop plants (e.g., wheat, maize, ryegrass) [44,45], and these crops usually serve as an important food source for cattle. In addition, animal feeds may contain some perfluorinated chemicals [24]. Moreover, PFAAs can bioaccumulate and biomagnify in terrestrial and aquatic food chains, thus more contaminants will accumulate in animals of upper trophic levels [29,46,47]. The occurrence of PFAAs in water has been reported frequently, and tap water is even regarded as a major route of exposure to PFOA for adults in Korea [21,40,46]. Air sources of PFAAs are many, such as from long-range transport, degradation of volatile precursors, atmospheric deposition, and direct emissions from manufacturing and industry [6,32,46]. PFAAs and their precursors in indoor air/dust do exist, although their concentrations are relatively low, and this surely contributes to the daily intake of these contaminants [22,48].

**Figure 5:**
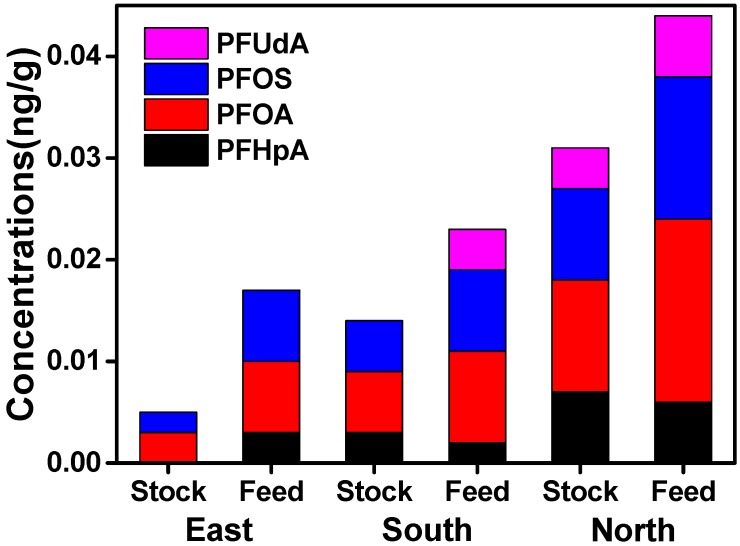
Concentrations (ng/g, wet weight) of PFAAs in commercially-fed/home-raised cattle muscle in Xinjiang (most liver samples lacked rearing information were not considered in this study). East: *n*_stock_ = 8; *n*_feed_ = 15; South: *n*_stock_ = 10; *n*_feed_ = 24; North: *n*_stock_ = 18; *n*_feed_ = 42.

The differences between PFAA levels in commercial feed and home-raised livestock may exist, due to living and eating habits causing different exposures to specific sources of these compounds, but there is no evidence for this yet. However, levels of PFAAs in eggs produced from differently raised chickens have been investigated [43], and home produced eggs were more contaminated because the chickens were raised outside and picked their food, worms or insects freely from the soil. By contrast, commercially raised chickens have less contact with the outdoor environment. In the present study, we compared the potential contribution of these two modes of feeding to PFAA exposure in cattle muscle, and found a completely opposite result, as shown in Figure 5. Although home-raised cattle have longer exposure to the outside environment, they mainly eat grass and their food sources are rather simple. In contrast, commercially raised cattle are exposed to more pollution sources via their diverse intake of feeds. In order to achieve rapid short-term fattening, they are given higher portions of animal feeds mixed with blood meal and fish meal, thus the mixed feeds may contribute to higher PFAA exposure [41].

### 3.5. Dietary Intake and Health Risk Assessment in Xinjiang

**Figure 6:**
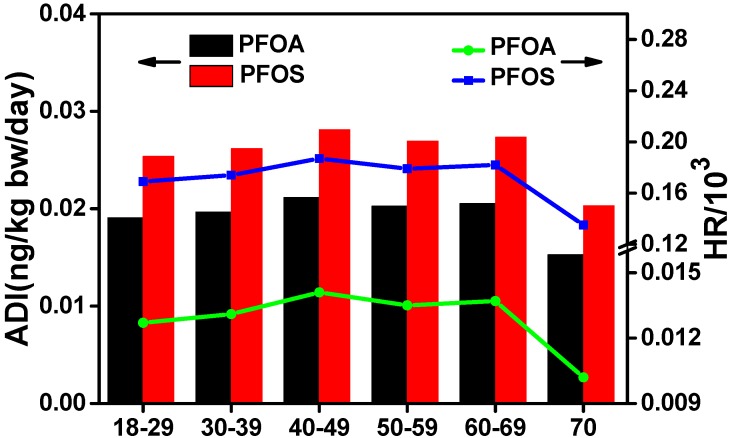
Average daily intake and hazard ratios of PFOS and PFOA in six age groups of Xinjiang.

In this survey, we made a hypothesis that beef muscle was the only consumed animal meat and beef liver was the only viscera intake. The health risks were assessed using the total meat/liver consumption data, and in this case, the estimated HR would be the highest exposure levels. PFOS and PFOA exposures though muscle and liver were examined for five parameters including region, urban/rural environment, gender, ethnicity, and age, and the results are shown in Table 2 and Figure 6. The highest ADI and HR values of these two compounds corresponded to northern Xinjiang (ADI_PFOS_: 0.039 ng/kg bw/day, ADI_PFOA_: 0.034 ng/kg bw/day; HR_PFOS_: 0.257 × 10^−3^, HR_PFOA_: 0.023 × 10^−3^) (Table 2). These ADI values are similar to another Xinjiang report on milk and yogurt (ADI_PFOS_: 0.032 ng/kg bw/day, ADI_PFOA_: 0.022 ng/kg bw/day) [5]. The daily PFAA dose has also been estimated in several countries such as Germany and Korea at about 10–100-fold higher than in the present study [21,44]. It is worth noting that only two food items were analyzed in our study, whereas a wide range of food items including fish, meat, vegetables, fruit, eggs, and milk were estimated in other countries. The ADIs of PFOS and PFOA for other groups were in the following order: urban > rural, male > female, Han Chinese ≈ others > Uighur. The age-related PFOS and PFOA exposure patterns were similar and increased with age and then decreased, as is shown in Figure 6. The highest amounts of these two compounds were found in the 40–49 years age group (ADI_PFOS_: 0.021 ng/kg bw/day, ADI_PFOA_: 0.028 ng/kg bw/day) and the lowest (ADI_PFOS_: 0.015 ng/kg bw/day, ADI_PFOA_: 0.020 ng/kg bw/day) in the elderly (> 70 years). Diet composition is an important factor for intake, and higher consumption of dairy products and fish/shellfish constitutes the main source of exposure in young children (1–6 years) in Korea [21].

**Figure 7:**
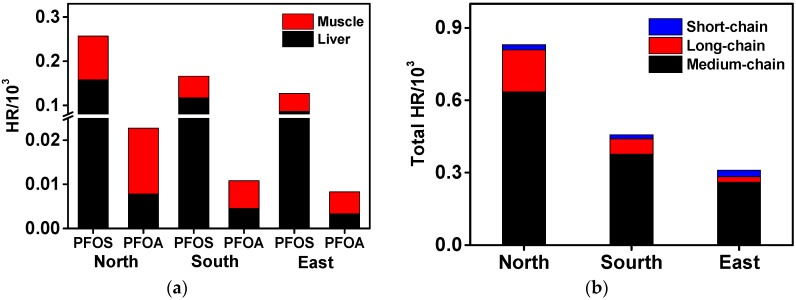
Hazard ratios of PFOA, PFOS, and ∑PFAAs in three parts of Xinjiang. (**a**) Contribution of muscle and liver to PFOS/PFOA exposure; (**b**) Contribution of different chain lengths of PFAAs to total PFAAs exposure.

Muscle was the major contributor to HR_PFOA_; however, liver mostly contributed to HR_PFOS_ (Figure 7a). To assess potential health risks of total PFAAs in Xinjiang adults through intake of these two foods, the total HR of PFAAs (HR_PFAAs_) was initially calculated and is presented in Figure 7b. In addition to PFOA and PFOS, other substances lack reference doses, thus HI_PFAAs_ were calculated using PFOS as the reference, which is only a rough estimation. It is worth mentioning that the bioaccumulation potential and toxicity of PFAAs increases with the chain length [18], thus for short-chain PFAAs, the health risk might be overestimated. However, it would be underestimated for long-chain PFAAs. Obviously, medium-chain PFAAs are the main contributors. The highest HR_PFAAs_ was calculated to be 0.837 × 10^−3^ (northern Xinjiang) (Figure 7b), which is far less than 1, suggesting that there is extremely small risk for the Xinjiang population via consumption of beef products.

## 4. Conclusions

The pollution status and health risk assessment of various PFAAs in animal meat samples of Xinjiang were systematically investigated for the first time. In summary, PFAA contamination was widespread in cattle samples, especially in liver, although the pollution levels were lower than in most other studies. PFOS, which is overwhelmingly observed in liver, and PFOA, were the dominant contaminants. In terms of regional variation, total PFAAs decreased in the following order: north > south > east. In terms of structure, they decreased in the following order: medium-chain > long-chain > short-chain. The results of source analysis showed that many factors could cause PFAA contamination, and feeding modes have a great influence on exposure. Remarkably, the risk ratios were much lower than 1 in all cases and suggest that no imminent damage to the health of the Xinjiang population via beef intake exists. Nevertheless, more categories of food are expected to be analyzed in Xinjiang, with the aim of comprehensively estimating PFAA exposure in humans, and several uncertainties (such as dietary preference, job/occupation/income, ventilation, and variations in dust level) should be taken into account. A wider age range, including toddlers, children, adolescents, and adults, should be considered to monitor and protect the health of different age brackets. Additionally, further studies are expected to investigate PFAAs found in the serum and plasma of humans, because their levels could better represent the degree of contamination, especially in the Xinjiang population.

## Figures and Tables

**Table 1:** The concentrations of perfluoroalkyl acids (PFAAs, ng/g, wet weight) in beef muscle and beef liver from Xinjiang.

Analytes	Muscle (*n* = 176)					Liver (*n* = 117)				
	Min.	Max.	Mean ± SE	Median	DF. (%)	Min.	Max.	Mean ± SE	Median	DF. (%)
PFBA	ND					ND	0.511	0.032 ± 0.00067		21
PFBS	ND					ND	0.320	0.020 ± 0.00061		19
PFPeA	ND					ND	0.734	0.003 ± 0.00001		3
PFHxA	ND					ND	0.253	0.016 ± 0.00021		12
PFHxS	ND					ND				
PFHpA	ND	0.061	0.004 ± 0.00001		17	ND	0.803	0.056 ± 0.00172		39
PFOA	ND	0.103	0.011 ± 0.00007		24	ND	1.532	0.264 ± 0.00896	0.221	75
PFOS	ND	0.082	0.008 ± 0.00003		30	ND	4.221	0.617 ± 0.05131	0.439	82
PFNA	ND					ND	1.243	0.184 ± 0.00691	0.056	56
PFDA	ND					ND	0.847	0.106 ± 0.00326	0.061	58
PFUdA	ND	0.066	0.003 ± 0.00002		8	ND	2.523	0.277 ± 0.02267	0.181	73
PFDoA	ND					ND	0.266	0.007 ± 0.00016		9
PFTrDA	ND					ND	0.532	0.036 ± 0.00112		21
∑PFAAs			0.026		47			1.632		94

Min.: minimum; Max.: maximum; DF.: detection rate; ND: concentration was below the LOD; SE: standard error.

**Table 2:** The consumption data, average daily intake, and hazard ratios of PFOS and PFOA for various groups (region, urban/rural, gender, and ethnicity).

Group		DC_muscle/liver_ (g/day)	ADI (ng/kg bw/day)		HR/10^3^	
			PFOA	PFOS	PFOA	PFOS
Region	North	75.0 (1.9) ^a^	0.034	0.039	0.023	0.257
	South	63.0 (1.6)	0.016	0.025	0.011	0.165
	East ^b^	75.0 (1.9)	0.013	0.019	0.008	0.127
Urban/rural	Urban	90.0 (2.3)	0.026	0.035	0.018	0.234
	Rural	62.0 (1.6)	0.018	0.024	0.012	0.161
Gender	Male	71.0 (1.8)	0.021	0.028	0.014	0.185
	Female	64.0 (1.6)	0.019	0.025	0.013	0.167
Ethnicity	Han Chinese	69.0 (1.7)	0.020	0.027	0.014	0.179
	Uighur	66.0 (1.7)	0.019	0.026	0.013	0.172
	Others	70.0 (1.8)	0.021	0.027	0.014	0.182

DC_muscle/liver_: daily consumption values of muscle/liver; ADI: average daily intake; HR: hazard ratios; ^a^ muscle (liver); ^b^ DC_muscle/liver_ of northern Xinjiang was used.

## Supplementary Materials

The following are available online at www.mdpi.com/1660-4601/14/9/970/s1, Table S1: The MS optimize parameters of 13 PFAAs and 2 internal standards, Table S2: Linear equation, correlation coefficient, LODs, LOQs, recovery and RSDs of 13 PFAAs in beef muscle and beef liver.
