# Supplementary material for: Occurrence, Distribution, and Risk Assessment of Perfluoroalkyl Acids (PFAAs) in Muscle and Liver of Cattle in Xinjiang, China
Source: Int J Environ Res Public Health. 2017 Aug 28;14(9):970. doi: 10.3390/ijerph14090970 (PMC5615507; doi:10.3390/ijerph14090970)
Supplement: Supplementary file 1 [file ijerph-14-00970-s001.pdf]

International Journal of
Environmental Research and Public Health

MDPI

Supplementary Files

Occurrence, Distribution, and Risk Assessment of Perfluoroalkyl Acids (PFAAs) in Muscle and Liver of Cattle in Xinjiang, China

Gehui Wang, Jianjiang Lu*, Zhenni Xing, Shanman Li, Zilong Liu, Yanbin Tong

Table S1. The MS optimize parameters of 13 PFAAs and 2 internal standards.

Compounds	Parent ion (m/z)	Daughter ion (m/z)	Retention time (min)	Fragment (V)	Collision energy (V)	Internal standard
PFBA	213	169*/93	1.15	70/70	3/15	MPFOA
PFPeA	263	219*/113	1.35	70/70	5/15	MPFOA
PFHxA	313	269*/119	1.70	70/70	3/15	MPFOA
PFHpA	363	319*/169	2.44	70/70	5/10	MPFOA
PFOA	413	369*/168.9	3.91	70/70	4/9	MPFOA
PFNA	463	419*/219	5.67	70/70	5/10	MPFOA
PFDA	513	469*/269	6.87	70/70	5/15	MPFOA
PFUdA	563	519*/269	7.83	70/70	5/20	MPFOA
PFDoA	613	569*/169	8.69	70/70	4/24	MPFOA
PFTrDA	663	619*/169	9.47	70/70	5/25	MPFOA
PFBS	299	99*/80	2.04	70/70	30/40	MPFOS
PFHxS	399	90*/80	4.89	70/70	40/48	MPFOS
PFOS	499	99*/80	7.48	70/70	48/72	MPFOS
MPFOA	417	372*/172	3.91	70/70	4/9	--
MPFOS	503	99*/80	7.48	70/70	48/72	--

*Quantitative ion pair.

Table S2. Linear equation, correlation coefficient, LODs, LOQs, recovery, and RSDs of 13 PFAAs in beef muscle and beef liver.

Analyte	Beef muscle						Beef liver					
	Linear equation	R^2	LOD (ng/g)	LOQ (ng/g)	Recovery[a] (%, n=6)	RSD (%, n=6)	Linear equation	R^2	LOD (ng/g)	LOQ (ng/g)	Recovery[a] (%, n=6)	RSD (%, n=6)
MPFOA					90.4	3.2					81.5	4.1
MPFOS					82.3	3.7					78.6	3.3
PFBA	y=547.02x	0.9979	0.023	0.077	110.5	7.1	y=911.79x	0.9986	0.015	0.050	80.9	5.2
PFPeA	y=720.80x	0.9918	0.050	0.167	103.6	5.8	y=1437.07x	0.9959	0.027	0.090	88.2	4.7
PFHxA	y=940.20x	0.9982	0.061	0.203	100.1	6.5	y=2270.31x	0.9959	0.010	0.033	86.5	5.1
PFHpA	y=862.44x	0.9937	0.031	0.103	91.5	4.6	y=1928.18x	0.9972	0.030	0.100	91.6	4.3
PFOA	y=921.27x	0.9998	0.021	0.070	106.2	6.2	y=1844.85x	0.9984	0.017	0.057	85.4	4.1
PFNA	y=1259.96x	0.9975	0.019	0.063	115.4	7.3	y=1551.80x	0.9975	0.008	0.027	104.1	3.5
PFDA	y=1300.95x	0.9991	0.017	0.057	108.6	5.7	y=1651.67x	0.9992	0.014	0.047	94.3	3.0
PFUdA	y=1486.35x	0.9981	0.037	0.123	103.8	8.3	y=1850.06x	0.9985	0.004	0.013	101.8	6.2
PFDoA	y=1526.18x	0.9986	0.012	0.040	93.6	5.5	y=2898.38x	0.9983	0.007	0.023	84.2	4.5
PFTrDA	y=1260.73x	0.9964	0.012	0.040	73.8	8.9	y=1737.77x	0.9978	0.003	0.010	90.4	5.6
PFBS	y=301.25x	0.9914	0.040	0.133	75.3	9.4	y=540.40x	0.9953	0.013	0.043	100.6	5.7
PFHxS	y=201.77x	0.9951	0.061	0.203	84.7	7.8	y=318.15x	0.9978	0.036	0.120	110.0	4.4
PFOS	y=220.77x	0.9976	0.013	0.043	88.2	4.6	y=254.27x	0.9978	0.009	0.030	103.6	6.3

[a] The mean recovery at 0.02, 0.5, 1 ng/g spiked levels; R^2: correlation coefficient; LOD: the limits of detection; LOQ: the limits of quantification; RSD: relative standard deviation.
